# Efficacy of PBTZ169 and pretomanid against Mycobacterium avium, *Mycobacterium abscessus*, *Mycobacterium chelonae*, and *Mycobacterium fortuitum* in BALB/c mice models

**DOI:** 10.3389/fcimb.2023.1115530

**Published:** 2023-03-22

**Authors:** Luyao Zheng, Xueting Qi, Weiyan Zhang, Hong Wang, Lei Fu, Bin Wang, Xi Chen, Xiaoyou Chen, Yu Lu

**Affiliations:** ^1^ Department of Pharmacology, Beijing Key Laboratory of Drug Resistance Tuberculosis Research, Beijing Tuberculosis and Thoracic Tumor Research Institute, Beijing Chest Hospital, Capital Medical University, Beijing, China; ^2^ Tuberculosis Department, Beijing Tuberculosis and Thoracic Tumor Research Institute, Beijing Chest Hospital, Capital Medical University, Beijing, China; ^3^ Infectious Diseases Department, Beijing Ditan Hospital, Capital Medical University, Beijing, China

**Keywords:** non-tuberculous mycobacteria, pretomanid, PBTZ169, murine model, BALB/c mice

## Abstract

**Objectives:**

We aimed to evaluate the activity of PBTZ169 and pretomanid against non-tuberculous mycobacteriosis (NTM) *in vitro* and *in vivo*.

**Methods:**

The minimum inhibitory concentrations (MICs) of 11 antibiotics, against slow-growing mycobacteria (SGMs) and rapid-growing mycobacteria (RGMs) were tested using the microplate alamarBlue assay. The *in vivo* activities of bedaquiline, clofazimine, moxifloxacin, rifabutin, PBTZ169 and pretomanid against four common NTMs were assessed in murine models.

**Results:**

PBTZ169 and pretomanid had MICs of >32 μg/mL against most NTM reference and clinical strains. However, PBTZ169 was bactericidal against *Mycobacterium abscessus* (3.33 and 1.49 log10 CFU reductions in the lungs and spleen, respectively) and *Mycobacterium chelonae* (2.29 and 2.24 CFU reductions in the lungs and spleen, respectively) in mice, and bacteriostatic against Mycobacterium avium and *Mycobacterium fortuitum*. Pretomanid dramatically decreased the CFU counts of *M. abscessus* (3.12 and 2.30 log10 CFU reductions in the lungs and spleen, respectively), whereas it showed moderate inhibition of *M. chelonae* and *M. fortuitum*. Bedaquiline, clofazimine, and moxifloxacin showed good activities against four NTMs *in vitro* and *in vivo*. Rifabutin did not inhibit *M. avium* and *M. abscessus* in mice.

**Conclusion:**

PBTZ169 appears to be a candidate for treating four common NTM infections. Pretomanid was more active against *M. abscessus*, *M. chelonae* and *M. fortuitum* than against *M. avium*.

## 1 Introduction

Multiple studies worldwide have shown that the prevalence of non-tuberculous mycobacteria (NTM) disease has been increasing over the last two decades (Ratnatunga et al., 2020). The clinical treatment regimens for these diseases are usually a combination of three or more drugs including macrolides for 18–24 months (Daley et al., 2020), but the treatment success rate is only 50%–66% on average (Raju et al., 2016; Diel et al., 2018), and there is a probability of relapse or conversion to drug resistance (Griffith et al., 2007). In addition, the high all-cause mortality rate and poor adherence to long-course therapy with injectable agents mean that new effective oral agents for safer and more effective treatment of NTM infections are required (Fleshner et al., 2016; Novosad et al., 2017; Mourad et al., 2021).

**Figure 1:**
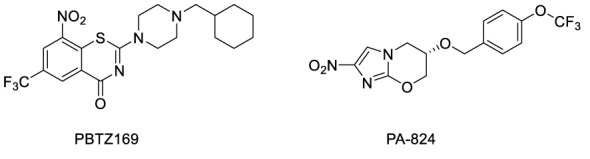
Structures of PBTZ169 and pretomanid(PA-824).

A straightforward strategy is to search for candidates against NTM among antibacterial or anti-tuberculosis (TB) drugs that are currently being developed, which is the most common approach in current anti-NTM drug screening studies. PBTZ169 (**Figure 1**) is a benzothiazinone compound that is less cytotoxic and shows significantly better efficacy at lower concentrations than the lead compound BTZ043 in a chronic TB murine model (Makarov et al., 2014). Pretomanid (**Figure 1**) is a nitroimidazooxazine antibacterial drug approved for pulmonary treatment-intolerant, non-responsive multidrug-resistant, or extensively drug-resistant TB in 2019 (Keam, 2019). These two anti-TB agents have good activities against *Mycobacterium marinum *(*M. marinum*) in zebrafish (Makarov et al., 2014; Dalton et al., 2017); however, their antibacterial activities against other species that cause NTM, especially in animal models, have not been reported.

In this work, in preliminary testing for drug activity, minimum inhibitory concentrations (MICs) were determined for 11 drugs (or compound) including PBTZ169 and pretomanid against NTM reference strains. Next, the *in vitro* antibacterial activities of four drugs with better activities and PBTZ169 and pretomanid in the preliminary assessment against NTM clinical strains (*M. avium*, *M. abscessus* and *M. fortuitum*) were investigated further. Because the potency observed *in vitro* for NTM often does not translate to an equivalent clinical efficacy against pulmonary infection, this study evaluated the *in vivo* antibacterial activities of the agents in murine models. We explored several new active agents that can be used to treat four common forms of NTM, providing a theoretical basis for the selection of clinical treatment options.

## 2 Materials and methods

### 2.1 Drugs and compounds

Isoniazid and rifampicin were purchased from Sigma-Aldrich. Dexamethasone, CLR, BDQ, CFZ, MOFX, ofloxacin, RFB, PBTZ169 and pretomanid were purchased from Biochempartner Co., Ltd. LZD was purchased from Ark Pharm, Inc.

### 2.2 Strains

*M. tuberculosis* (H37Rv), *M. kansasii* (ATCC 12478), *M. marinum* (ATCC 927), *M. gordonae* (ATCC 14470), *M. scrofulaceum* (ATCC 19981), *M. avium* (ATCC 25291), *M. intracellulare* (ATCC 13950), *M. xenopi* (ATCC 19250), *M. abscessus* (ATCC 19977), *M. chelonae* (ATCC 14472), *M. fortuitum* (ATCC 6841), and *M. gilvum* (ATCC 43909) were obtained from the National Clinical Laboratory on Tuberculosis, Beijing Chest Hospital. The *M. tuberculosis* and non-tuberculous mycobacterial strains were grown in Middlebrook 7H9 broth (Difco) supplemented with 0.2% (vol/vol) glycerol, 0.05% Tween 80, and 10% (vol/vol) oleic acid-albumin-dextrose-catalase (Becton-Dickinson).

### 2.3 MIC measurements

MICs were determined by a microplate-based alamarBlue assay (Xu et al., 2019). Briefly, bacteria (2 × 10^5^ CFU/mL) were added to wells, yielding a final testing volume of 200 μL. The plates were incubated at 37°C. On day 3 (for RGMs) or 7 (for SGMs) of incubation, 20% Tween 80 (12.5 μL) and alamarBlue (20 μL) were added to all wells. After incubation at 37°C for another 24 h, the fluorescence was measured at an excitation wavelength of 530 nm and an emission wavelength of 590 nm. The MIC was defined as the lowest concentration eliciting a reduction in fluorescence of ≥90% relative to the mean fluorescence of replicate drug-free controls. *M. tuberculosis* H37Rv was used as a drug-susceptible control.

### 2.4 Development of mouse model

Seventy female BALB/c mice (18 to 20 g) were inoculated intravenously in the tail vein. Dexamethasone (5 mg/kg) was freshly prepared in carboxymethyl cellulose (CMC), and the treatment began 1 week prior to infection, was stopped 6 days after infection, administered daily, and was not administered on the day of infection. To achieve an implantation of 4.0–5.0 log_10_ CFU in the lungs of a mouse, a primary *M. abscessus* culture in the exponential phase, with A_600nm_ of 1.00–1.20, was used to prepare a suspension by diluting to a calculated A_600nm_ of 0.1 in normal saline. CLR was administered 5 days a week for 3 weeks after stopping dexamethasone. Sacrifices were performed 1 day post infection (D-6), on the day of CLR administration (D0), and then at D7, D14, D21, and D28 post infection.

*M. abscessus*, *M. chelonae*, and *M. fortuitum* mouse models were established as described above. One hundred and eighty mice were randomly divided into four groups and infected with four types of mycobacteria. Five mice infected with different bacteria were humanely killed 1 day after infection and on the day of treatment initiation. The number of bacteria implanted in the lungs and spleen (or kidneys) were determined by plating serial dilutions of tissue homogenates on nutrient 7H10 agar. The remaining 35 mice in each group were randomly allocated among seven groups, including one untreated control group, and six groups were treated with BDQ, CFZ, RFB, MOFX, pretomanid, or PBTZ169 monotherapy.

### 2.5 Experimental chemotherapy trials

Treatment was initiated 7 days after infection. For treating the mice, BDQ, CFZ, RFB, MOFX, PBTZ169 and pretomanid were suspended in 0.5% (wt/vol) CMC, whereas MOFX was diluted with normal saline. All antimicrobial agents were given for 28 days (7 times weekly) by gavage, and mice in the control group were administered CMC. The dosages for each treatment, per kilogram of body weight, were 20 mg/kg RFB (Singh et al., 2021), 25 mg/kg BDQ (Zhang et al., 2019), 25 mg/kg CFZ (Baijnath et al., 2015), 25 mg/kg PBTZ169 (Makarov et al., 2014), 100 mg/kg MOFX (Andrejak et al., 2015) and 200 mg/kg pretomanid, identical to the effective dosages of these antimicrobial agents against *M. tuberculosis* infection of mice. The dose selection of pretomanid was based on our previous experiments (data not shown), in which 100 mg/kg pretomanid (Xu et al., 2019) was ineffective against NTM in mice, so 200 mg/kg was used in this experiment.

Efficacy was assessed based on the lung, spleen, or kidney (mice infected with *M. fortuitum*) CFU counts. Comparisons to determine significant effects of antibiotic compounds are in reference to the D28 untreated control. Mice were sacrificed 3 days after the last day of treatment to reduce carryover effects. The organs were aseptically removed and homogenized. Suspensions were made up to 1 mL for each organ. At least four serial 10-fold dilutions of the suspension were performed on 0.5% charcoal-containing selective 7H10 plates. The plates were incubated for up to 1 (for RGMs) or 4 (for SGMs) weeks at 37°C before the final CFU counts were determined.

### 2.6 Statistical analysis

Organ CFU counts were log-transformed before analysis, and mean CFU counts were compared by one-way analysis of variance with Dunnett’ s *post hoc* test to control for multiple comparisons. The Mann-Whitney test was used to test for significance on nonnormally distributed CFU data. All analyses were performed with GraphPad Prism version 5 (GraphPad, San Diego, CA). A *P* value of 0.05 was considered significant.

## 3 Results

### 3.1 *In vitro* antimycobacterial activity

As shown in **Table 1**, first-line antitubercular drugs, isoniazid and rifampicin, had limited activities against NTM *in vitro*, especially against *M. avium*, *M. abscessus*, *M. chelonae*, and *M. fortuitum*, and they were partly used as controls. Clarithromycin (CLR) is a baseline drug for the treatment of NTM lung disease, and our study showed that CLR had good activities against all NTMs except *M. chelonae* (24.922 μg/mL).

**Table 1:** MICs of 11 drugs (or compound) against different mycobacterial species.

Mycobacterial species	MICs (μg/mL)										
	INH	RFP	CLR	BDQ	CFZ	RFB	LZD	MOFX	OFLX	Pretomanid	PBTZ169
*Mtb*, H37Rv	0.05	0.06	7.24	0.03	0.41	0.039	0.51	0.06	0.34	0.12	0.001
*M. kansasii*	0.11	3.22	0.21	0.02	0.12	<0.001	0.91	0.07	0.29	1.71	<0.001
*M. marinum*	0.49	>32	1.88	0.03	0.21	<0.016	0.58	1.89	7.60	>32	<0.001
*M. gordonae*	>32	0.05	0.13	0.01	0.30	0.05	0.39	0.06	0.27	>32	0.002
*M. scrofulaceum*	>32	0.02	0.09	0.02	1.28	0.01	3.93	0.06	0.21	>32	>32
*M. avium*	>32	30.22	3.27	0.21	0.82	0.07	1.51	3.96	12.82	>32	>32
*M. intracellulare*	1.88	1.32	0.93	0.06	0.49	0.06	4.85	0.47	4.69	>32	>32
*M. xenopi*	>32	0.12	0.02	1.96	0.21	<0.016	0.31	<0.016	0.05	3.84	<0.016
*M. abscessus*	>32	>32	1.86	0.46	6.91	3.91	35.87	6.00	23.29	>32	>32
*M. chelonae*	>32	>32	24.92	1.33	5.80	5.86	60.15	15.32	>32	>32	>32
*M. fortuitum*	9.39	>32	2.98	0.74	12.64	1.71	>64	0.23	0.94	>32	0.01
*M. gilvum*	3.37	0.05	0.21	0.12	1.92	0.002	0.52	0.10	0.23	>32	<0.001

*Isoniazid(INH), rifampicin(RFP), clarithromycin(CLR), bedaquiline(BDQ), clofazimine(CFZ), rifabutin(RFB), Linezolid(LZD), moxifloxacin(MOFX), ofloxacin(OFLX).

Of the remaining nine drugs (or compound), PBTZ169 showed good activities against *M. kansasii*, *M. marinum*, *M. gordonae*, *M. xenopi*, *M. fortuitum* and *M. gilvum* (MICs of <0.016 μg/mL), but had no activity against other 5 NTMs (MICs of >32 μg/mL) against the 11 NTMs. Pretomanid exhibited poor *in vitro* activities against most NTMs we tested, only *M. kansasii* and *M. xenopi* were sensitive to pretomanid. Bedaquiline (BDQ) and rifabutin (RFB) had the lowest range of MIC values for the antibiotics we tested. The MICs for BDQ against 11 reference strains ranged from 0.01 to 1.96 μg/mL, which were higher than those for RFB (MICs of <0.001 to 5.86 μg/mL). For the fluoroquinolones, the activity of moxifloxacin (MOFX) against NTM was superior to that of ofloxacin. Furthermore, clofazimine (CFZ) and linezolid (LZD) had different activities against NTM in RGMs and SGMs, with MICs of 0.12 to 4.51 μg/mL against the RGMs and 0.39 to 5.81 μg/mL against the SGMs, respectively. CFZ was moderately active against all four RGMs (MICs of 1.92to 12.64μg/mL), whereas LZD was ineffective against all three RGMs, except *M. gilvum* (MIC of 0.52 μg/mL).

BDQ, CFZ, RFB, and MOFX were the four most active of the 11 antibiotics, so their *in vitro* activities against three common NTM clinical strains, *M. avium*, *M. abscessus*, and *M. fortuitum*, were tested. Because we did not collect clinical strains of *M. chelonae*, no evaluation was performed. Although the NTMs were insensitive to PBTZ169 and pretomanid *in vitro*, these compounds were also included in subsequent studies. The results suggested that BDQ, CFZ, RFB, and MOFX also had good *in vitro* activities against *M. avium*, *M. abscessus*, and *M. fortuitum* clinical strains (**Table 2** and **Supplementary Table 1**).

**Table 2:** MIC ranges of clinical strains of *M. avium*, *M. abscessus* and *M. fortuitum for* BDQ, CFZ, RFB, MOFX, PBTZ169 and Pretomanid.

Antimicrobial	MIC range(μg/mL)			
	*M. avium* (n=9)	*M. abscessus subsp. abscessus* (n=13)	*M. abscessus subsp. massiliense* (n=7)	*M. fortuitum* (n=1)
CLR	2.21 - >32	1.57 - >32	0.08 - >32	1.70
BDQ	0.03 - 0.47	0.41 - 3.82	0.09 - 3.21	<0.016
CFZ	0.82 - 3.74	2.43 - 15.10	2.56 - 13.77	1.80
RFB	0.04 - 0.96	3.78 - 14.90	2.79 ->32	0.40
MOFX	0.19 - 5.87	7.73 - >32	1.65 - >32	<0.016
Pretomanid	>32	>32	>32	>32
PBTZ169	>32	>32	>32	<0.016

### 3.2 *In vivo* efficacy in mice

To assess the efficacy of antibiotics, we established murine models of *M. avium*, *M. abscessus*, *M. chelonae*, and *M. fortuitum* disease. Although the anti-NTM activities of PBTZ169 and pretomanid were poor *in vitro*, they significantly reduced the *M. marinum* load in zebrafish (Makarov et al., 2014; Dalton et al., 2017); thus, we evaluated the anti-NTM activities of BDQ, CFZ, RFB, MOFX, PBTZ169, and pretomanid in mice.

**Figure 2:**
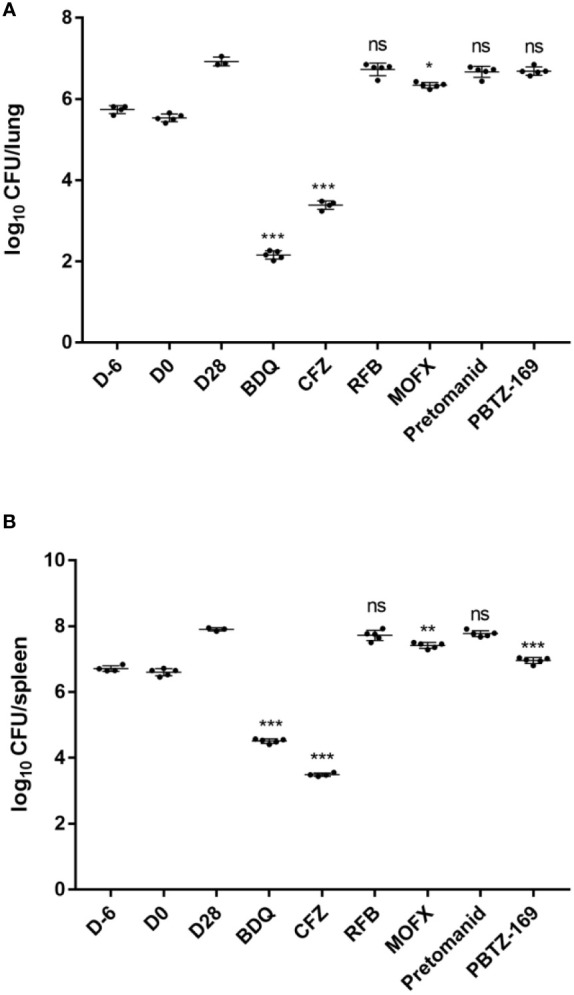
Mean number (log_10_) of CFU per lung **(A)** and spleen **(B)** in various groups of mice infected with *M. avium*. Two mice failed to infect due to operational reasons, bacteria did not enter the tail vein, showing thickened tail redness and swelling, and mice weighed significantly higher than other members of this group (one each from CMC and CFZ group). One mouse from CMC group died during the treatment period. D-6 represents the day after infection, D0 represents the start of drug treatment in mice, and D28 represents twenty-eight days after drug treatment in mice. *P <0.05; **P <0.01; ***P <0.001; “ns” indicates no statistically significant difference between the two groups.

The bacterial load of *M. avium* in mouse organs continued to rise throughout the experimental period (**Figure 2**, **Table 3**). At 1 day post infection, all mice were implanted with 5–6 log_10_ CFU of *M. avium* in the lungs and spleen. At the end of treatment, the intrapulmonary bacterial load was ~7 log_10_ CFU and the bacterial load in the spleen was ~8 log_10_ CFU. PBTZ169 and MOFX showed weak bacteriostatic activities against *M. avium*, reducing the bacterial load by <1 log_10_ CFU. In addition, pretomanid and RFB did not have an inhibitory effect on *M. avium*. BDQ and CFZ showed excellent antimicrobial activities against *M. avium* in the lungs and spleen, and they reduced the bacterial load in the lungs by 4.76 and 3.53 log_10_ CFU and in the spleen by 3.40 and 4.42 log_10_ CFU, respectively.

**Table 3:** *M.avium* burden in the lungs and spleens of mice.

	D-6	D0	D28	BDQ	CFZ	RFB	MOFX	Pretomanid	PBTZ169
Mean log_10_ CFU/lung ± SD	5.74 ± 0.11	5.52 ± 0.09	6.92 ± 0.11	2.16 ± 0.10	3.39 ± 0.10	6.73 ± 0.15	6.34 ± 0.07	6.67 ± 0.14	6.69 ± 0.10
Mean log_10_ CFU/spleen ± SD	6.72 ± 0.09	6.61 ± 0.11	7.91 ± 0.05	4.52 ± 0.07	3.49 ± 0.05	7.72 ± 0.16	7.42 ± 0.09	7.77 ± 0.09	6.96 ± 0.08

All groups began with equivalent lung implantation burdens. D-6 refers to 1 day after infection, D0 refers to initiation of drugs treatment in mice.

**Figure 3:**
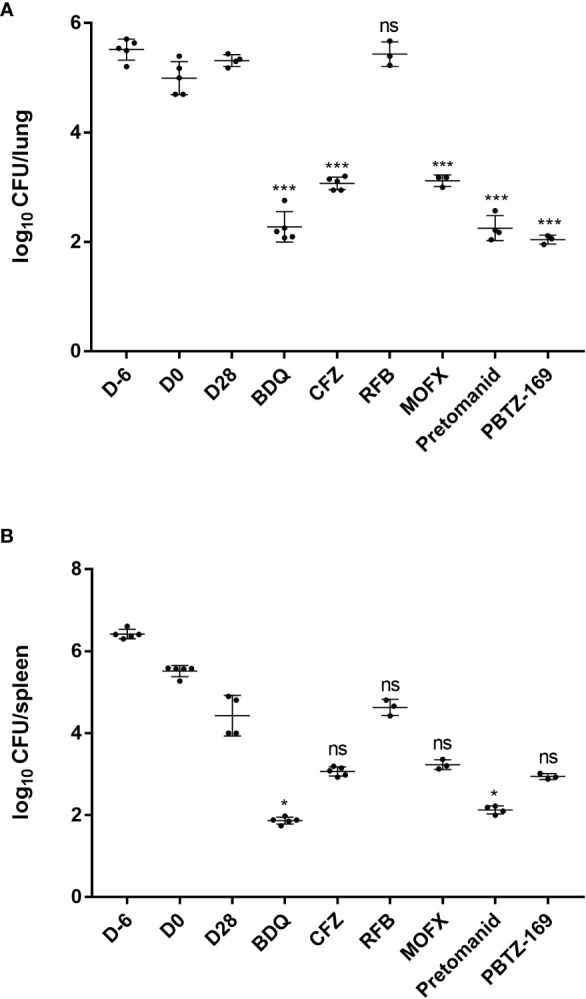
Mean number (log_10_) of CFU per lung **(A)** and spleen **(B)** in various groups of mice infected with *M. abscessus. *Four mice failed to infect due to operational reasons (1 each from RFB, MOFX, pretomanid and PBTZ169 group). Three mice died during the treatment period (1 each from CMC, RFB, and MOFX group).*P <0.05; **P <0.01; ***P <0.001; “ns” indicates no statistically significant difference between the two groups.

In the mice infected with *M. abscessus*, as with previous results, a high bacterial load was maintained in the lungs throughout the infection and treatment periods. However, the bacterial load in the spleen showed a continuous decrease; thus, the antibacterial effect of the drug present in the spleen was partly attributed the clearance of mycobacteria by the mice’s immune system (**Figure 3**; **Table 4**). Throughout the experiment, three mice (5.5%) developed characteristic spinning disease, in which the mice held their heads to one side and often shook and twitched their heads, and when suspended by their tails, they rotated vigorously. In contrast to the *in vitro* sensitization results, both PBTZ169 and pretomanid had strong antimicrobial activities in the lungs (3.33 and 3.12 log_10_ CFU reduction, respectively), and pretomanid had strong antimicrobial activity (2.30 log_10_ CFU reduction) in the spleen too, whereas PBTZ169 reduced the bacterial load by only 1.49 log_10_ CFU. BDQ showed good activities against *M. abscessus in vitro* and *in vivo* in the lungs (3.09 log_10_ CFU reduction) and in the spleen (2.56 log_10_ CFU reduction). The antibacterial activity of CFZ and MOFX in the lungs (2.30 and 2.25 log_10_ CFU reduction) was better than that in the spleen (1.37 and 1.20 log_10_ CFU reduction). Although *M. abscessus* was sensitive to the RFB *in vitro*, RFB had no antimicrobial activity in mouse lungs or spleen.

**Table 4:** *M.abscessus* burden in the lungs and spleens of mice.

	D-6	D0	D28	BDQ	CFZ	RFB	MOFX	Pretomanid	PBTZ169
Mean log_10_ CFU/lung ± SD	5.51 ± 0.19	4.95 ± 0.33	5.37 ± 0.06	2.28 ± 0.28	3.07 ± 0.14	5.73 ± 0.30	3.12 ± 0.16	2.25 ± 0.23	2.04 ± 0.08
Mean log_10_ CFU/spleen ± SD	6.42 ± 0.12	5.50 ± 0.14	4.43 ± 0.50	1.87 ± 0.08	3.06 ± 0.11	5.63 ± 0.20	3.23 ± 0.14	2.13 ± 0.10	2.94 ± 0.07

All groups began with equivalent lung implantation burdens.

**Figure 4:**
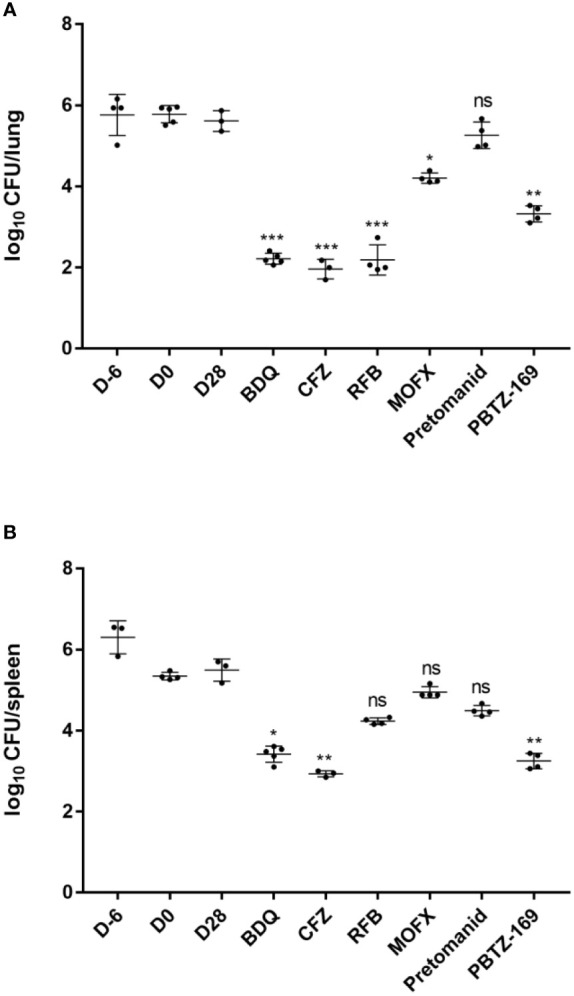
Mean number (log_10_) of CFU per lung **(A)** and spleen **(B)** in various groups of mice infected with *M. chelonae*. Six mice failed to infect due to operational reasons(one each from CFZ, RFB, MOFX and PBTZ169 group, and two from CMC). Two mice died during the treatment period (one each from CFZ and pretomanid group). *P <0.05; **P <0.01; ***P <0.001.

Unlike *M. abscessus*, *M. chelonae* remained at high levels in the lungs and spleen throughout the infection and treatment periods (**Figure 4**; **Table 5**). Throughout the experiment, PBTZ169 and pretomanid were equally ineffective against *M. chelonae in vitro*, but unlike the *M. abscessus* infection group, pretomanid had little *in vivo* antimicrobial activity, whereas PBTZ169 showed good antimicrobial activities in both lungs and spleen (2.29 and 2.24 log_10_ CFU reduction). The activities of BDQ and CFZ against *M. chelonae* in mice in the lungs (3.40 and 3.68 log_10_ CFU reduction) and spleen (2.07 and 2.56 log_10_ CFU reduction) were similar to those for *M. abscessus*. However, unlike the *M. abscessus* group, RFB had better activities against *M. chelonae* (3.43 log_10_ CFU reduction in the lungs and 1.25 log_10_ CFU reduction in the spleen). MOFX was active only in the lungs (1.41 log_10_ CFU reduction), and had weak activity in the spleen.

**Table 5:** *M. chelonae* burden in the lungs and spleens of mice.

	D-6	D0	D28	BDQ	CFZ	RFB	MOFX	Pretomanid	PBTZ169
Mean log10 CFU/lung ± SD	5.61 ± 0.45	5.75 ± 0.22	5.62 ± 0.26	2.22 ± 0.14	1.94 ± 0.24	2.19 ± 0.37	4.21 ± 0.13	5.26 ± 0.33	3.33 ± 0.20
Mean log10 CFU/spleen ± SD	6.20 ± 0.58	5.34 ± 0.09	5.49 ± 0.28	3.42 ± 0.20	2.93 ± 0.08	4.24 ± 0.08	4.95 ± 0.14	4.49 ± 0.13	3.25 ± 0.19

All groups began with equivalent lung implantation burdens.

**Figure 5:**
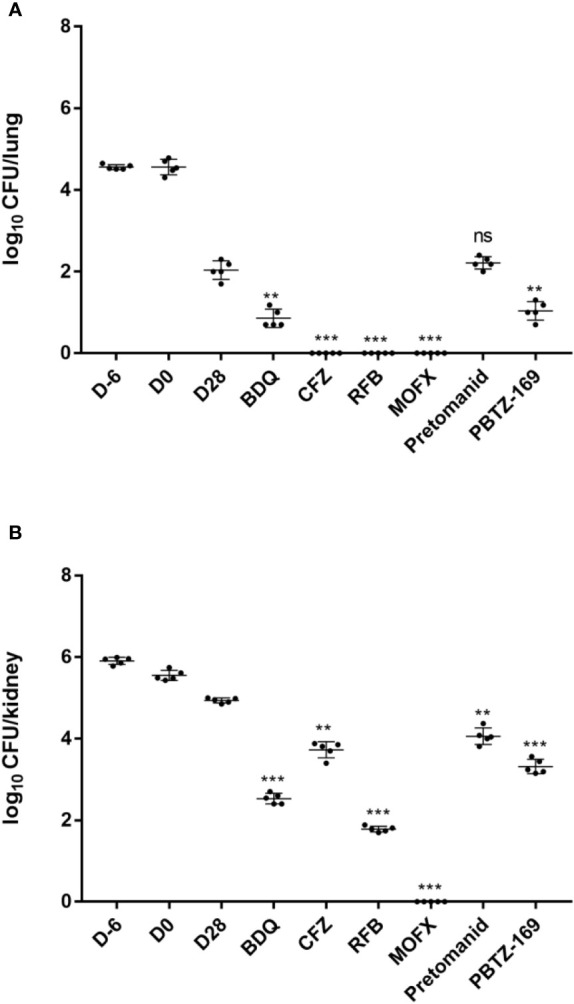
Mean number (log_10_) of CFU per lung **(A)** and kidney **(B)** in various groups of mice infected with *M. fortuitum*. No mice experienced procedural failure or mortality. *P <0.05; **P <0.01; ***P <0.001.

Almost half of the mice infected with *M. fortuitum* developed rotational disease (27/55, 49.1%), which was the highest of the four animal models we established. The bacterial load in the spleen was 4.49 ± 0.19 log_10_ CFU/spleen 1 day after *M. fortuitum* infection, whereas it decreased to 3.44 ± 0.13 log_10_ CFU/spleen on the day of treatment (data not shown). This was consistent with previous reports that *M. fortuitum* in the spleen in mice decreases over time until complete clearance (Parti et al., 2005). The bacterial load in the lungs was 4.56 ± 0.06 log_10_ CFU/lung on day 1 post infection, remained stable at 4.51 ± 0.19 log_10_ CFU/lung on the day of treatment, and decreased to 1.97 ± 0.20 log_10_ CFU/lung after 28 days of treatment, also consistent with the trend reported in the literature, but with a greater decrease in CFU. Although there was a significant decrease in intrapulmonary bacterial load in mice with obvious immune clearance involved, BDQ and PBTZ169 had moderate activities against *M. fortuitum* in mouse lungs, whereas RFB, CFZ, and MOFX were more active (**Figure 5A**; **Table 6**). Next, we analyzed the antibacterial activities of the drugs in mouse kidneys (**Figure 5B**; **Table 6**). Pretomanid showed only weak activity (0.88 log_10_ CFU reduction), whereas PBTZ169 exhibited moderately strong activity (1.62 log_10_ CFU). MOFX was the most active against *M. fortuitum*, whitch completely cleared the bacterial load from the kidneys. BDQ and RFB showed consistent activities *in vitro* and *in vivo*, and they exhibited bactericidal activities in the kidneys (2.41 and 3.15 log_10_ CFU reduction). CFZ cleared *M. fortuitum* from the lungs, but only inhibited the bacteria in the kidneys.

**Table 6:** *M.* fortuitum burden in the lungs and spleens of mice.

	D-6	D0	D28	BDQ	CFZ	RFB	MOFX	Pretomanid	PBTZ169
Mean log10 CFU/lung ± SD	4.56± 0.06	4.51± 0.19	1.97± 0.20	0.85± 0.22	0	0	0	2.21± 0.15	1.04± 0.23
Mean log10 CFU/kidney ± SD	5.91± 0.09	5.57± 0.12	4.94± 0.06	2.53± 0.13	3.70± 0.22	1.79± 0.06	0	4.06 ± 0.20	3.32± 0.18

All groups began with equivalent lung implantation burdens.

## 4 Discussion

Treatment regimens of NTM lung disease often require long-term therapy with multiple antimicrobial drug combinations (Gill et al., 2021). Even so, the global cure rate for NTM infection is comparable to or worse than that for multidrug-resistant TB, and recurrence or reinfection is common (Daley et al., 2020; Dartois and Dick, 2022). There is an urgent need to find effective oral agents to treat NTM. Therefore, this study determined the activities of some anti-TB drugs against NTM *in vitro* and vivo to provide new candidates for treating NTM.

*In vitro* activity assays showed that both PBTZ169 and pretomanid had MICs of >32 μg/mL against most NTM reference and clinical strains, whereas BDQ, CFZ, RFB, and MOFX had the lowest MICs. The inhibitory activities of these six drug (or compound) against the clinical isolates of the three most common species (*M. avium*, *M. abscessus*, and *M. fortuitum*) were consistent with the results from the reference strains. PBTZ169 is reported to be active against *M. marinum in vitro* with a MIC of 0.0003 μg/mL, whereas the MICs against *M. avium* and *M. abscessus* were >100 μg/mL (Chauffour et al., 2020). Similarly, PBTZ169 showed good activities against SGMs *M. kansasii*, *Mycobacterium gordonae*, and *M. xenopi*, yielding MICs of <0.016 μg/mL, and it was active against RGMs *M. fortuitum* and *M. gilvum*. According to previous studies, pretomanid was moderately active *in vitro* against *M. kansasii* (MIC_90_ of 8 mg/L), and was inactive against most NTMs (MIC_90_ of >32 mg/L) (Zhang et al., 2020). Similar to these reports, of the 11 NTMs we tested, only pretomanid was active *in vitro* against *M. kansasii* (MIC of 1.708 μg/mL) and *M. xenopi* (MIC of 3.842 μg/mL).

**Figure 6:**
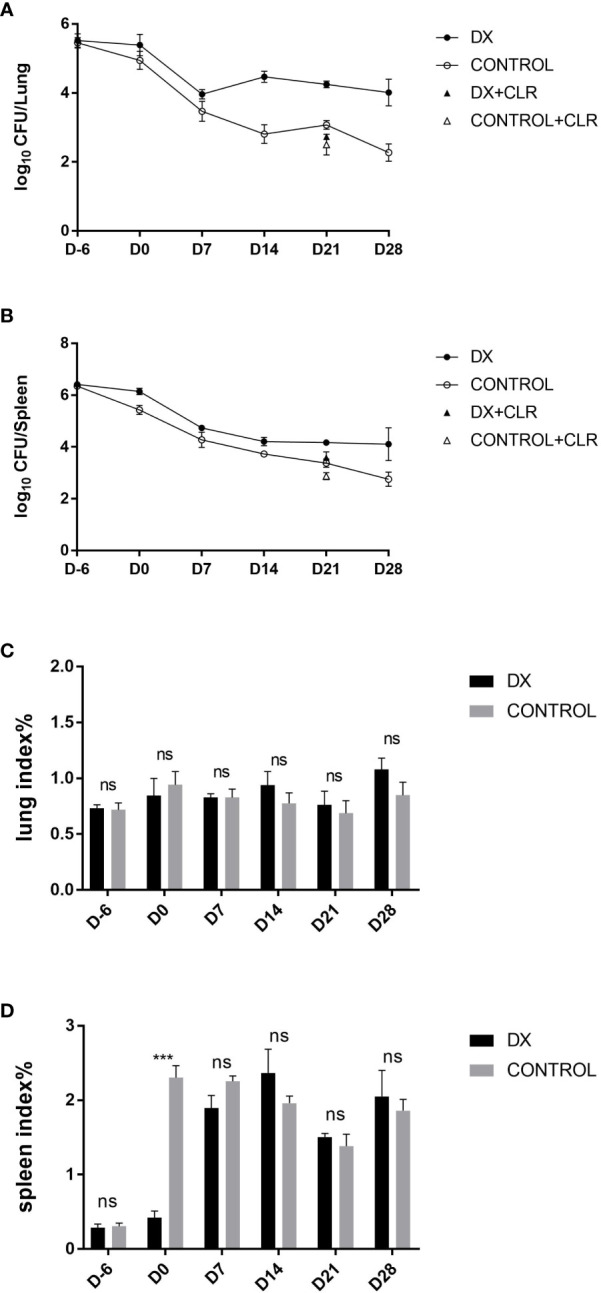
Mean number (log_10_) of CFU and organ indices in various groups of mice infected with *M. abscessus*. **(A, B)** represent *M. abscessus* burdens in the lungs and spleens of mice, and **(C, D)** represent organ indices in the lungs and spleens of mice, respectively. The organ indices is the ratio of the organ weight to whole body weight. Plot of mean lung/spleen log_10_ CFU. “DX” indicate mice treated with dexamethasone before and after infection, “CONTROL” indicate mice treated with CMC instead of dexamethasone. “DX+CLR or CONTROL+CLR” indicate mice given dexamethasone or CMC starting CLR treatment 7 days after infection and administered 5 days a week for a total of 3 weeks. D7, D14, D21 and D28 represents the 7th, 14th, 21st and 28th day after treatment in mice, respectively. “ns” indicates no statistically significant difference between the two groups. ***P<0.001.

Our previous study showed that BALB/c mice infected with aerosols of *M. abscessus* and *M. avium* did not maintain a certain bacterial load in the lung tissue (data not shown). Andréjak et al. reported that the effects of antibiotic treatment were most pronounced in BALB/c mice (Andrejak et al., 2015). We selected BALB/c mice as model animals, *M. abscessus* was injected *via* the tail vein of the mice, and dexamethasone was given to suppress immunity 1 week before and 1 week after infection to observe the stability of the model. The results showed that the administration of dexamethasone before drug treatment did not affect the body weight, lung index, and spleen index of mice during the later drug evaluation (weeks 1–4). The changes in CFU were more pronounced after administration of CLR, and a high bacterial load was maintained 1–4 weeks after infection (**Figure 6**; **Tables 7**, **8**), which could be used for evaluating the *in vivo* (pulmonary) anti-*M. abscessus* effect of the drugs.

**Table 7:** *M. abscessus* burden in the lungs of mice receiving different corticosteroid treatments.

Treatment Group	Mean log_10_ CFU/Lung ± SD					
	D-6	D0	D7	D14	D21	D28
DX	5.51 ± 0.19	5.41 ± 0.31	3.97 ± 0.14	4.44 ± 0.17	4.16 ± 0.04	4.02 ± 0.38
DX+CLR					2.84 ± 0.28	
Control	5.45 ± 0.12	4.94 ± 0.26	3.58 ± 0.14	2.83 ± 0.27	3.00 ± 0.05	2.27 ± 0.25
Control+DX					2.41 ± 0.34	

**Table 8:** *M. abscessus* burden in the spleens of mice receiving different corticosteroid treatments.

Treatment Group	Mean log_10_ CFU/Spleen ± SD					
	D-6	D0	D7	D14	D21	D28
DX	6.42 ± 0.11	6.09 ± 0.06	4.77 ± 0.05	4.19 ± 0.18	4.14 ± 0.05	4.11 ± 0.64
DX+CLR					3.59 ± 0.22	
UN	6.35 ± 0.10	5.37 ± 0.21	4.39 ± 0.17	3.77 ± 0.05	3.54 ± 0.15	2.76 ± 0.27
UN+DX					2.87 ± 0.13	

The results showed that *M. avium*, *M. abscessus*, and *M. chelonae* sustained replication in the BALB/c mice, and high bacterial loads were maintained in the lungs and spleen. *M. fortuitum* maintained a high bacterial load in the kidneys, although the bacteria were almost completely cleared from the spleen and eventually only a bacterial load of 1.97 log_10_ CFU/lung remained in the lungs. There is no explanation of the renal tropism yet, but we propose that *M. fortuitum* was cleared from the lungs because it often causes skin and wound infections, and intrapulmonary infections are less common. Furthermore, *M. fortuitum* is much less toxic than the other three NTMs and the mouse immune system clears it more easily.

PBTZ169 and pretomanid have very poor *in vitro* properties. Nevertheless, in mice, PBTZ169 had good *in vivo* antibacterial activities against all four tested NTMs, especially the three RGMs, and pretomanid significantly reduced the *M. abscessus* load in all organs and showed moderate inhibition of *M. chelonae* and *M. fortuitum* in the spleen. Additionally, BDQ, CFZ, and MOFX showed good activities against the four NTMs. CFZ and fluoroquinolones are recommended in the guidelines for treating NTM (Daley et al., 2020; Gill et al., 2021; Lange et al., 2022). In these experiments, the *in vivo* and *in vitro* anti-NTM activities of these two drugs were both excellent and showed a correlation. Unlike the results of MICs, RFB inhibited only *M. chelonae* and *M. fortuitum* incidentalis in mice, and was ineffective against *M. avium* and *M. abscessus*.

The results showed the PBTZ-169 and pretomanid activities *in vitro* and *in vitro* were inconsistent, which suggests that although *in vitro* screening is a key first step, *in vivo* evaluation for NTM drugs seems to be more important. PBTZ169 showed great differences in activity against different NTMs *in vitro*, although the DprE1 enzyme has been reported to be highly conserved in mycobacterium (Shi et al., 2018), the amino acid type at critical codons such as Cys387 still impacts mycobacterial susceptibility to PBTZ169 (Batt et al., 2012; Shi et al., 2018). In this experiment, PBTZ169 showed excellent activity against NTMs in mice. Martínez et al. demonstrated that PBTZ69 significantly reduced Nocardia brasiliensis in macrophages at sub-bactericidal concentrations (Gonzalez-Martinez et al., 2015). So it is likely that PBTZ169 increases the bactericidal effect of macrophages by some way, thus showing excellent inhibition of all four NTMs in mice. There are 2 possible explanations for the differential *in vitro* activity of pretomanid against different mycobacterium species, which are the absence of targets or cannot be activated. However, pretomanid significantly reduced *M. abscessus* burden in mice. Pretomanid produces active NO when interacting with mycobacteria, it increases the conversion of *M. abscessus* from smooth morphotype(SM) to rough morphotype (RM) and produces more cytokines (Nandanwar et al., 2021) to induce T cell-mediated immune activation (Kam et al., 2022). This was indirectly demonstrated by our finding of large numbers of rough colonies on 7H10 solid medium cultured from tissue homogenate of mice treated with pretomanid alone when CFU counts were performed. Studies have shown that the RM of *M. avium* does not have increased sensitivity to antibiotics (Saito and Tomioka, 1988) and similarly pretomanid showed no additional killing activity against *M. avium* in mice in our study.

Mechanistically, BDQ can weaken or abrogate the proton motive force (PMF) of bacteria, making bacteria more sensitive to environmental changes (pH or other drugs). Furthermore, BDQ interferes with the function of F_1_F_0_-ATP synthase, affecting the energy supply of the bacterial body (Rao et al., 2001; Rao et al., 2008; Hards et al., 2015). PMF is necessary for the survival of mycobacteria, including *M. tuberculosis*, *M. smegmatis*, and *M. marinum* (Rao et al., 2001; Liu et al., 2020), which is also consistent with our experimental results that BDQ has good antibacterial activities against the four common NTMs, both *in vivo* and *in vitro*. Importantly, our experiments demonstrate the potential use of BDQ for the treatment of NTM pulmonary diseases and we found a good correlation between its ex vivo and *in vivo* activities. During our experiments, in the first 2 weeks of administration, the mice in the BDQ group lost weight, their fur lost its luster and became ruffled, and they showed reduced activity, similar to the control group. In the latter 2 weeks, the mice in the BDQ group gradually gained weight, their coats regained their luster, and they were active again, as reported previously (Koul et al., 2014). One theory is that BDQ affects bacterial PMF, but early PMF depletion leads to bacterial respiratory activation that compensates for depletion (Hards et al., 2015). Another theory is that BDQ may be resisted in M. abscessus by the induction of dormant regulators and activation of the ATP production pathway, thereby maintaining bacterial viability during initial drug exposure and meaning that BDQ shows a delayed bactericidal effect (Koul et al., 2014). Furthermore, the redistribution of the accumulated drug can provide an additional bactericidal effect. More experiments are needed to verify this.

Our study also has some shortcomings. First, BALB/c mice do not produce specific tissue lesions, such as granulomas, and thus cannot be used to evaluate the ability of drugs to penetrate lesions. Second, the model had a short testing cycle and did not assess the long-term stability of the murine model. The aim of this study was to provide a preliminary screen for active drugs against NTM, and the effect of antibiotic treatment was most pronounced in BALB/c mice compared with other mouse strains, so we believed that it was more appropriate to use this mouse strain to establish a model. The acute infection model was used because this experiment was an initial screening of drugs.

In conclusion, our experiments showed that PBTZ169 is a potentially effective agent for the treatment of *M. avium*, *M. abscessus*, *M. chelonae*, and *M. fortuitum.* In addition, pretomanid may be an excellent candidate for treating *M. abscessus* pneumonia and further clinical trial studies should be performed.

## Data availability statement

The raw data supporting the conclusions of this article will be made available by the authors, without undue reservation.

## Ethics statement

The animal study was reviewed and approved by Animal Ethics Committee of the Beijing Chest Hospital-Affiliate of Capital Medical University.

## Author contributions

YL, XYC and LZ designed the study. LZ performed *in vitro* experiments. LZ, XQ, WZ, HW, LF, BW and XC performed animal experiments. LZ performed analysis and drafted the original manuscript. All authors re-viewed and YL edited the manuscript. The study was supervised by YL and XYC. All authors have read the manuscript and have approved its submission for publication.

## Supplementary material

Supplementary Table 1 MICs of the drugs (or compounds) against clinical strains of NTM.
