# Supplementary material for: Efficacy of PBTZ169 and pretomanid against Mycobacterium avium, Mycobacterium abscessus, Mycobacterium chelonae, and Mycobacterium fortuitum in BALB/c mice models
Source: Front Cell Infect Microbiol. 2023 Mar 22;13:1115530. doi: 10.3389/fcimb.2023.1115530 (PMC10106926; doi:10.3389/fcimb.2023.1115530)
Supplement: Supplementary Table 1 — MICs of the drugs (or compounds) against clinical strains of NTM. [file Table_1.pdf]

SUPPLEMENTARY TABLE 1 MICs of the drugs (or compounds) against clinical strains of NTM

Mycobacterial species		MICs (μg/mL)						
		CLR	BDQ	CFZ	RFB	MOFX	Pretomanid	PBTZ169
M. avium	*Reference*	3.27	0.21	0.82	0.07	0.19	＞32	＞32
	2—8	2.21	0.04	1.67	0.11	0.95	＞32	＞32
	2—9	3.53	0.05	1.96	0.10	0.90	＞32	＞32
	2—11	3.86	0.16	1.88	0.10	0.92	＞32	＞32
	2—15	3.84	0.03	1.95	0.04	0.88	＞32	＞32
	2—16	3.41	0.07	1.90	0.96	3.34	＞32	＞32
	8—1	5.38	0.11	3.74	0.11	0.65	＞32	＞32
	8—9	30.65	0.47	0.82	0.47	0.97	＞32	＞32
	8—11	3.56	0.03	1.73	0.08	2.95	＞32	＞32
	8—15	＞32	0.03	3.20	0.17	5.87	＞32	＞32
M. abscessus subsp. abscessus	*Reference*	2.76	0.46	6.91	3.91	6.00	＞32	＞32
	0021	11.48	0.41	7.16	7.08	7.73	＞32	＞32
	0023	6.13	0.46	2.43	5.74	9.07	＞32	＞32
	M-111	＞32	3.11	13.64	14.51	＞32	＞32	＞32
	M-124	＞32	2.84	13.22	4.71	29.00	＞32	＞32
	M-163	6.09	0.90	7.93	6.38	＞32	＞32	＞32
	M-165	1.98	1.94	14.41	3.86	＞32	＞32	＞32
	M-168	＞32	3.82	14.19	7.78	＞32	＞32	＞32
	M-172	＞32	0.95	7.82	7.75	＞32	＞32	＞32
	M-237	1.57	3.80	14.94	14.72	＞32	＞32	＞32
	M-253	7.91	1.22	14.51	3.78	＞32	＞32	＞32
	M-269	29.92	2.78	15.10	14.90	＞32	＞32	＞32
	M-288	6.65	1.84	14.65	7.09	29.91	＞32	＞32
	M-323	＞32	1.74	14.94	3.91	15.49	＞32	＞32
M. abscessus subsp. massiliense	*0019*	0.08	0.09	3.48	5.50	1.65	＞32	＞32
	0032	0.08	0.10	5.08	7.38	2.01	＞32	＞32
	M-119	1.11	0.57	6.91	6.33	20.28	＞32	＞32
	M-126	＞32	3.21	12.52	2.79	26.10	＞32	＞32
	M-131	2.59	2.59	2.56	＞32	13.90	＞32	＞32
	M-133	1.98	2.34	13.77	15.36	＞32	＞32	＞32
	M-234	1.69	1.31	13.49	22.01	＞32	＞32	＞32
	M-242	1.70	3.27	13.32	14.66	＞32	＞32	＞32
	M-274	0.75	0.61	13.23	15.20	3.78	＞32	＞32
M. fortuitum	*Reference*	27.98	0.74	12.64	1.71	0.23	＞32	0.008
	XA99	1.70	＜0.016	1.80	0.40	＜0.016	＞32	＜0.016
